# Assessment of technical adequacy of sacral lateral branches cooled radiofrequency neurotomy

**DOI:** 10.1016/j.inpm.2022.100069

**Published:** 2022-02-17

**Authors:** Yakov Vorobeychik, Bunty Shah, Vitaly Gordin, David Giampetro, Chachrit Khunsriraksakul, To-Nhu Vu

**Affiliations:** a Penn State Health Milton S. Hershey Medical Center, Penn State College of Medicine, Department of Anesthesiology and Perioperative Medicine, HU32, 500 University Drive, P.O. Box 850, Hershey, PA, 17033-0850, USA; b Penn State College of Medicine, Hershey, PA, USA

**Keywords:** Sacroiliac joint pain, Radiofrequency ablation, Middle cluneal nerves, Buttock hypoesthesia, Neurotomy

## Abstract

**Objective:**

There were two primary objectives of the study: 1. assessment of the association between diagnostic sacral lateral branches (SLB) blocks and the ensuing numbness in the middle cluneal nerves (MCN) distribution, irrespective of whether the patients had positive or negative responses to blocks. 2. If the consistency of this causal relationship was established, we wanted to investigate a further correlation - hypoesthesia from local anesthetic blocks vs. hypoesthesia from radiofrequency neurotomy (RFN) vs. outcomes.

**Design:**

This is a prospective observational study of sixty consecutive patients with sacroiliac (SI) joint complex pain and failure of previous intraarticular SI joint injection. The patients who had two positive diagnostic SLB blocks defined as ≥ 75% reduction in NRS scores were treated with cooled RFN of the L5 dorsal ramus and S1–S3 lateral branches. The patients were interviewed and evaluated at a one-month post-neurotomy follow-up appointment. Seven patients were also evaluated at a six-month follow-up visit after the procedure.

**Methods:**

The primary outcomes of the study were absence/presence of post-procedural buttock hypoesthesia after diagnostic blocks and absence/presence of post-procedural buttock hypoesthesia at one month after a cooled RFN procedure. The secondary outcome measures related to the effectiveness of this procedure and included: pre- and post-procedure NRS scores; ODI scores initially, and at post RFN follow-up; analgesic consumption initially, and at one-month RFN follow-up; patient satisfaction with the cooled RFN treatment. A procedure was considered categorically successful if the patient gained ≥50% pain relief and was satisfied with its results.

**Results:**

81/84 (96.4%; 95% CI [89.9%, 99.3%]) of the diagnostic SLB blocks lead to temporary sensory deficit to pinprick in the MCN distribution. If the block was positive, 58/58 (100.00%; 95% CI [93.8, 100.00%]) of the procedures led to hypoesthesia. For negative diagnostic blocks, 3/26 (11.5%; 95% CI [2.4%, 30.2%]) procedures lead to no hypoesthesia. The buttock hypoesthesia persisted in all patients with successful cooled RFN one month after this intervention. Among the patients with unsuccessful RFN, only 2/9 (22.2%, 95%CI [2.8%, 60.0]) still had hypoesthesia, but the rest of this group had no sensory deficit on pinprick examination. At 6-months follow-up buttock hypoesthesia had no association with the success of the procedure.

The patients' average NRS scores decreased from baseline 7.1 (SD 1.7) to 4.3 (SD 3.3) at 1-month follow-up after RFN. Categorical success, based on ≥50% pain relief coupled with patients' satisfaction, was achieved in 12/21 (57.1%; 95% CI [34.0%, 78.2%]) of the subjects. Average ODI percentage score decreased from 41.7% (SD 15.1%) to 31.8% (SD 17.8%) at the primary endpoint of the study.

**Conclusion:**

MCNs provide regular and clinically detectable innervation to the skin area overlaying posterior-medial aspects of the gluteus maximums muscle. Therefore, any technically accurate diagnostic block, irrespective of whether the patients have positive or negative responses, should result in the development of hypoesthesia in the area supplied by the MCNs. Immediately after the completion of the diagnostic procedure, the adequacy of the block should be tested. Absence of hypoesthesia suggests that the block may have been technically inadequate. Numbness in the buttock area innervated by the MCNs may serve as a marker of an adequately performed RFN procedure. If this procedure is unsuccessful in patients who do not develop post-neurotomy numbness in the area supplied by the MCNs, the failure of the intervention may stem from its inaccurate implementation rather than from its inherent ineffectiveness.

## 1 Introduction

The reported prevalence of sacroiliac joint complex (SIJC) pain, which includes the sacroiliac joint (SIJ) proper but also the posterior extraarticular structures, such as overlying posterior ligaments, tendons and regional muscles that support and cover the joint [1], depends on the criteria for positive diagnostic blocks used in different studies. One systematic review of 45 articles reported a 10%–33% range of SIJC pain among the patients with axial LBP below the L5 vertebral level for the studies utilizing >75% pain relief after dual diagnostic blocks [2]. The prevalence of SIJC pain is even higher in patients after lumbosacral fusion [3,4]. DePalma et al. [5] found that almost 43% of such patients had SIJP vs. only 18% of those who were not-fused, which means that this type of LBP was the most prevalent in patients who underwent lumbosacral fusion.

Numerous studies evaluated the efficacy and effectiveness of intraarticular SIJ steroid injections and demonstrated various degrees of success that most of the time did not exceed 3-months [2]. A proportion of patients with SIJC pain nonresponsive to intraarticular SIJ injection may have their pain generator located in the sacroiliac ligaments rather in the joint itself. Experimentally, pain induced by stimulating the sacroiliac ligaments, but not from distention of SI joint, was prevented by local anesthetic block of the sacral lateral branches (SLB) of the dorsal rami, possibly because the SIJ has both dorsal and ventral nerve supplies [6]. Interestingly, a recent randomized control trial compared the effectiveness of intraarticular fluoroscopy guided SIJ injections with a steroid and local anesthetic with non-image-guided injections targeting the extraarticular structures and found the former only slightly more effective [7].

In contradistinction to intraarticular injections, radiofrequency neurotomy (RFN), targeting the nerves supplying the SJIC may provide more sustained pain relief for properly selected patients. One of the most popular RFN techniques utilizes water cooled RF electrodes that allow creation of large lesions even if they are not placed parallel to the targeted nerves. Several observational studies reported the success rate from this procedure (defined ≥50% pain relief) ranging from 32 to 80% at 6-months after RFN [[8], [9], [10], [11], [12]]. Two more rigorous explanatory studies reported the six-month post-ablation success to be 58% in Cohen et al. [13] paper and 38% in Patel et al. [14] article. Both of these studies were thoroughly analyzed in the most recent review by Yang et al. [15].

Based on the currently available body of evidence, it is obvious that a large proportion of patients with SIJC pain do not gain a desirable outcome from cooled RFN of the SLB regardless of the rigor of selection criteria for this intervention. One of the plausible explanations for the failure of cooled RFN in some patients is the great anatomical variability of the SLB emerging from the sacral foramina at different sites and even at different depths [6,[16], [17], [18], [19]]. Therefore, unless the procedure is performed assiduously, some (or many) nerves innervating the SIJC may escape RFN lesions. Interestingly, the anatomy of the SLB may provide a means to evaluate the accuracy and adequacy of lesions created by cooled RFN of the SLB. Cadaveric studies showed that the middle cluneal nerves (MCN) that derive from the S1-3 SLB supply the skin overlying the postero-medial fifth of the gluteus maximus muscle [18,20,21]. Theoretically, blocking or ablating their parental nerves should result in hypoesthesia in the area supplied by MCN, thereby providing objective evidence of the technical accuracy of this procedure.

We had two primary objectives of this study. First, we assessed the association between diagnostic SLB blocks and the ensuing numbness in the MCN distribution, irrespective of whether the patients had positive or negative responses to blocks. Second, if the consistency of this causal relationship was established, we wanted to investigate a further correlation: hypoesthesia from local anesthetic blocks vs. hypoesthesia from RFN vs. outcomes.

## 2 Material and methods

The study was conducted at the ambulatory pain medicine clinic of a tertiary care academic medical center located in central Pennsylvania. Permission to conduct the study was granted by Hershey Medical Center (HMC) IRB (#00002420), and the study was registered on the Clinicaltrials.gov website (Registration # NCT02808962.) The patients' recruitment occurred between 2016 and 2021. Because of COVID-19 pandemic related restrictions on human research conduction at HMC, the study's activities were suspended for several months in 2020 and in the beginning of 2021.

The objective of the study was to determine whether the presence of post-procedural hypoesthesia in the buttock area innervated by the MCNs can be used as a marker of adequate performance of cooled radiofrequency neurotomy of the nerves supplying the posterior SIJC. Demonstrating the correlation between the success of this procedure and the presence of post-procedural buttock hypoesthesia will prove this hypothesis. Therefore, the primary outcomes of the study were absence/presence of post-procedural buttock hypoesthesia after diagnostic blocks and absence/presence of post-procedural buttock hypoesthesia at one month after a cooled RFN procedure. The secondary outcome measures related to the effectiveness of RFN and included: pre- and post-procedure Numeric Rating Scale (NRS) scores; Oswestry Disability Index (ODI) scores initially, and at post cooled RFN follow-up; analgesic consumption initially, and after cooled RFN follow-up; patient satisfaction with the cooled RFN treatment. A procedure was deemed categorically successful if the patient gained ≥50% pain relief and was satisfied with its results. Some patients were followed at six months' post-ablation, and both primary and categorical secondary outcomes of these patients were also reported.

Subjects for the present study were drawn from all patients referred to the clinic with low back pain by spine surgeons, neurosurgeons, and primary care physicians. The inclusion criteria were as follows: axial pain below the L5 vertebrae; pain duration of ≥6 months; three-day average NRS scores of ≥3/10; age greater than 18 years; failure of conservative treatment, including nonsteroidal anti-inflammatory medications and physical therapy; pain localized to the SIJ region; failure of injection of steroids into the SIJ to achieve adequate improvement. The patients who failed SIJ injections had at least three positive SIJ stress maneuvers, as described in previous studies [[22], [23], [24]]. The exclusion criteria were as follows: radicular pain; systemic infection or localized infection at the anticipated introducer entry site; pregnancy; allergy to lidocaine; individuals unable to consent; bleeding dyscrasias; non-English speaking patients; patients who were illiterate.

**Fig. 1:**
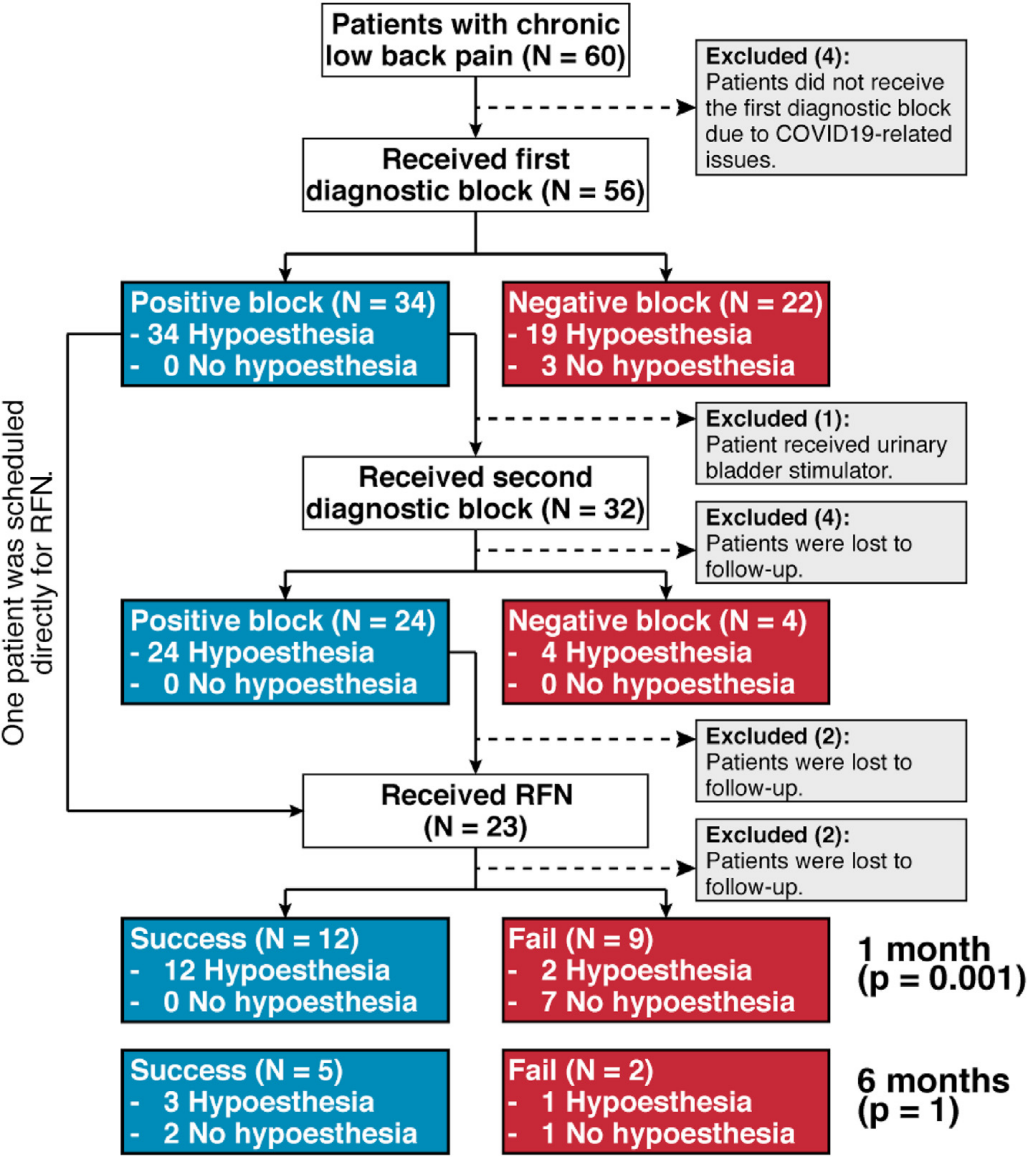
Cohort flow chart.

During the initial, screening visit, the patients underwent a routine physical examination, wherein the eligibility of the subjects for the study was determined. The participating physicians explained the study and obtained informed consent from the eligible patients. The baseline and post-neurotomy interviews at the patients' follow-up visits were conducted by a research associate who did not participate in the patients' treatment. Sixty out of approximately five hundred consecutively screened patients were recruited into the study (Fig. 1.) The baseline demographic data, as well as a 3-day average NRS score, ODI score, and analgesic consumption information were obtained during the initial visit. The participants were then scheduled for the set of two fluoroscopy-guided diagnostic blocks with 0.5 ​ml of 1% lidocaine of the lateral branches of S1, S2, and S3 dorsal rami nerves and of the dorsal ramus of the L5 nerve. The L5 dorsal ramus block was performed according to SIS practice guidelines [25], and S1, S2, and S3 lateral branch nerves were blocked using the technique described by Patel et al. [14]. A brief pinprick sensory exam of both buttock areas was performed after each block (regardless of laterality of local anesthetic injections), and the patients were given a pain diary to complete after the procedure to the point when the pain intensity reached the pre-procedure level. Because sensory testing for hypoesthesia after SLB blocks was conducted mere minutes post-procedure, true blinding of investigators could not be accomplished due to the visibility of needle entry sites on the subjects' skin. The results of the exam were documented in a binary fashion, i.e. “no sensory deficit” or “sensory deficit to pinprick in the buttock area”. Subjects who obtained ≥75% pain relief for the duration of action of the local anesthetic after both blocks underwent cooled RFN of the above mentioned nerves. 17-gauge cooled RF probes (Avanos Medical, Alpharetta, GA, USA) were used for the RFN. The technical aspects of this procedure were described previously [13,14,26], and this technique was replicated in our study. The patients were evaluated at one month after cooled RFN (primary endpoint of the study.) At that time, during a face-to-face interview, the following data were collected: 3-day average NRS scores, satisfaction with the treatment, ODI, and analgesic consumption. Physical evaluation, including sensory exam of the buttock areas was performed. Some of the patients whose procedures were successful were to be scheduled for the second follow-up at 6 months' post-intervention. However, the COVID-19 pandemic significantly disrupted the 6-month follow-up visits, and many patients were unable to keep their final appointments. Nonetheless, the primary and categorical outcomes of those patients who were able to appear at 6-month follow-up were also reported in this study.

## 3 Calculation

Statistical analyses were conducted in R-version 3.6 software. Descriptive statistics are presented as counts or proportions for categorical variables and as medians [quartile1 (Q1), quartile 3 (Q3)] for continuous variables. The chi-square test was used to assess the association between hypoesthesia and the success of RFN procedure, while Wilcoxson rank-sum test was used to assess differences for NRS and ODI at baseline and 1-month post-RFN. The confidence intervals for population proportions were estimated using an exact binomial confidence interval. A normal approximation confidence interval was not used due to the small number of successes/failures (<10).

## 4 Results

### 4.1 Demographics

Almost 500 subjects with axial low back pain below the L5 vertebral level were screened for recruitment, and the vast majority of them did not meet the inclusion criteria. Two patients declined to be enrolled in the study. Overall, sixty patients were consented to participate in the study (Fig. 1) Because clinical human research at HMC was intermittently suspended since the beginning of COVID-19 pandemic, some patients had to discontinue their participation at different stages of the study. There were more females than males enrolled (Table 1). The average baseline NRS score of the cohort was 7.5, and the average baseline ODI percentage score was 42% (31%, 55%). Eighteen of sixty patients were taking opioid analgesics.

**Table 1:** Demographics of the cohort.

Variable	Value
**Age** (Median [IQR]), N ​= ​60	60.00 [51.00, 70.00]
**Gender**, N ​= ​60	
Female	45
Male	15
**Pain duration**, N ​= ​57	
≤5 years	33
>5 years and ≤ 10 years	14
>10 years	10
**Baseline NRS** (Median [IQR]), N ​= ​58	7.50 [6.50, 8.50]
**Baseline ODI** (Median [IQR]), N ​= ​58	42.00% [30.50%, 54.75%]
**Lumbosacral fusion**, N ​= ​56	
Yes	9
No	39
Unsure	8
**Opioid consumption**, N ​= ​60	
Yes	18
No	42

Fifty-six patients received the first diagnostic block, and 32 progressed to the second one. One patient with a positive first block had to exit the study because she received a urinary bladder stimulator and was no longer considered to be an eligible candidate for RFN due to safety concerns. One patient, who reported 100% pain relief after the first diagnostic block, was scheduled directly for RFN bypassing the second block. Four patients had to withdraw from the study after the second block because of COVID-19 related issues. Out of 24 subjects with two positive diagnostic blocks, two dropped out and 23 (including the one who skipped the second block) received RFN treatment. One of these patients moved out of the area, and one patient was lost to follow-up. Seven of 12 patients who reported successful treatment at 1-month follow-up were also evaluated and interviewed at the 6-month follow up post-neurotomy.

### 4.2 Primary outcomes

**Fig. 2:**
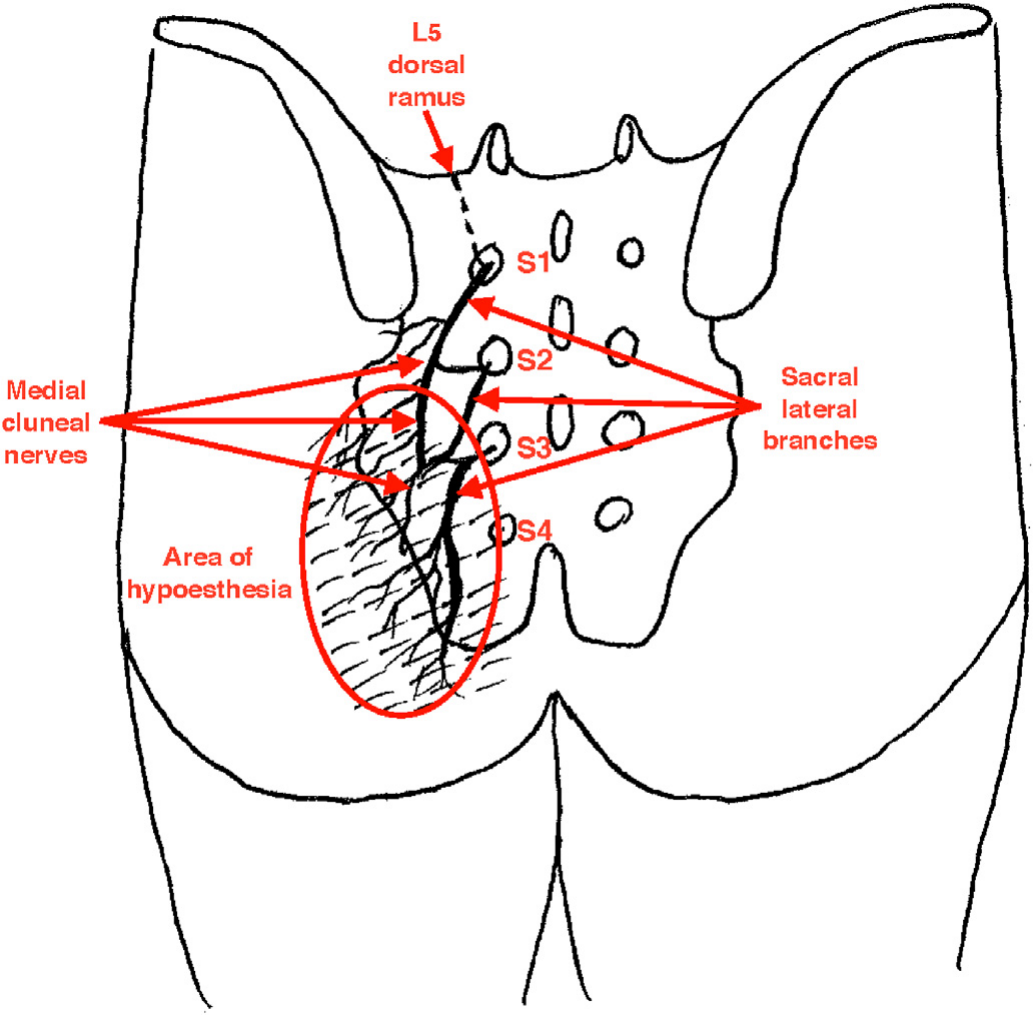
Schematic illustration of anesthesia zone developed after SLB diagnostic blocks and RFN.

The vast majority of diagnostic blocks resulted in temporary hypoesthesia in the buttock area innervated by the MCNs (Fig. 1) Consistently, in 81/84 (96.4%; 95% CI [89.9%, 99.3%]) of the procedures, the blocks lead to temporary sensory deficit to pinprick in just a few minutes after the injection (Fig. 2). If the block was positive, 58/58 (100.00%; 95% CI [93.8, 100.00%]) of the procedures led to hypoesthesia. For a negative diagnostic block, 3/26 (11.5%; 95% CI [2.4%, 30.2%]) procedures lead to no hypoesthesia. All study subjects developed sensory deficit in the buttock after the second diagnostic block, irrespective of the effect of the injection on the index pain.

The buttock hypoesthesia persisted in all patients with successful cooled RFN one month after this intervention. Among the patients with unsuccessful RFN, only 2/9 (22.2%, 95%CI [2.8%, 60.0]) still had hypoesthesia, but the rest of this group had no sensory deficit on pinprick examination. Only seven of twelve patients whose RFN was determined to be successful at one-month follow-up were evaluated at six months post neurotomy. At that time, buttock hypoesthesia had no association with the success of the procedure - no sensory deficit was found in 2/5 (40.0%, 95%CI [5.3%, 83.3%]) of patients with continuous success after the neurotomy and in one of two patients whose pain increased after the three months of initial improvement and reached the level of >50% of the pre-procedural baseline on NRS.

### 4.3 Secondary outcomes

**Fig. 3:**
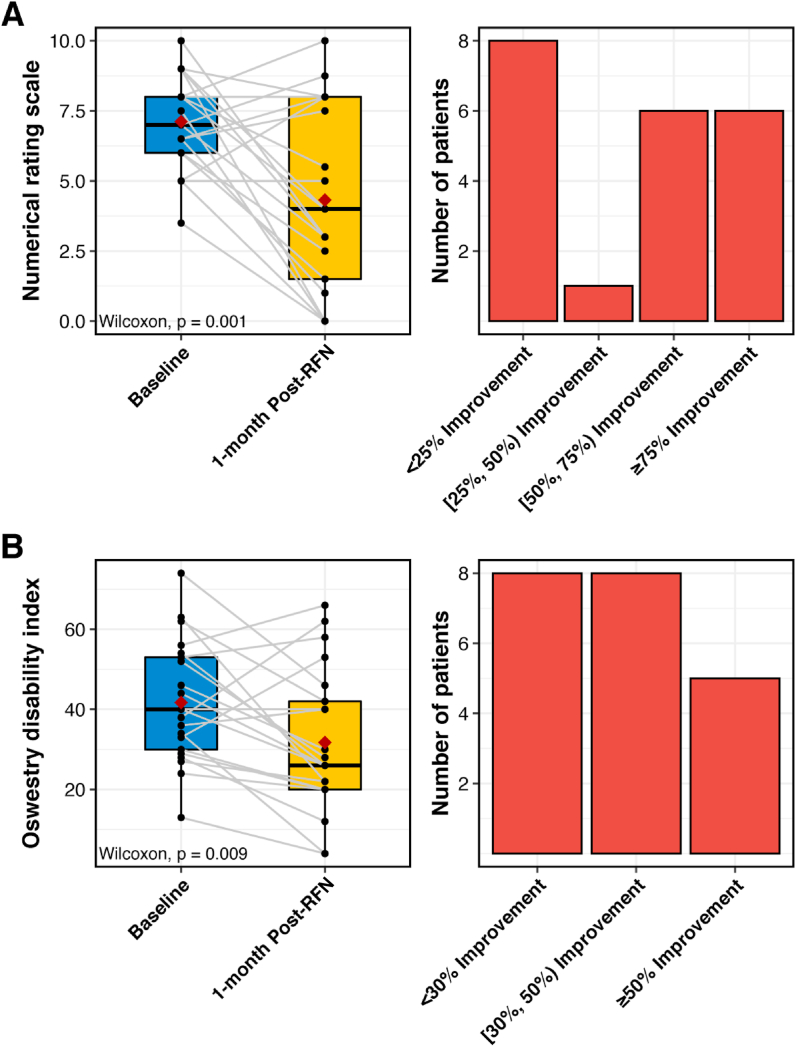
Secondary outcome measurements related to the effectiveness of RFN, including numerical rating scale (A) and Oswestry Disability Index (B). The analysis only included patients who have data available at baseline and 1-month post-RFN (N ​= ​21). Red dots inside the boxplot refer to the average values. Bolded lines inside the boxplot refer to the median values.

Analysis of the group data revealed that at 1-month follow-up after RFN, the patients' average NRS scores decreased from the baseline of 7.1 (SD 1.7) to 4.3 (SD 3.3), exceeding the minimal clinically important difference (MCID) of 2–2.5 points on NRS generally accepted for axial low back pain [[27], [28], [29]]. In order to overcome the limitations of the group scores, the number of patients who achieved various degrees of relief is presented in Fig. 3. Categorical success, based on ≥50% pain relief coupled with patient satisfaction, was achieved in 12/21 (57.1%; 95% CI [34.0%, 78.2%]) of the subjects (52,2%; 95% CI [31.8%, 72.6%] if the worst case scenario is applied.) Five out of seven patients (71.4%; 95% CI [29.0%, 96.3%]) who were evaluated at 6 months post RFN continued having ≥50% reduction in pain scores.

Average ODI percentage score decreased from 41.7% (SD 15.1%) to 31.8% (SD 17.8%) at the primary endpoint of the study. There is no single agreed-upon MCID for ODI with some investigators using the points change, while others use percentage change to determine this outcome measure [30]. Improvement by 30% was suggested by some as the cut point for MCID [31,32], and 50% improvement was advocated to be considered as a successful outcome [33]. In our study, 13/21 (61.9%), and 5/21 (23.8%) reached those benchmarks accordingly (Fig. 3.) Seven out of twenty-one patients who underwent RFN were taking opioid analgesics at baseline; two of them stopped 1-month post-RFN, and two others decreased the dose of these medications at 1-month follow-up.

## 5 Discussion

Our study is the first to prove that almost every patient develops an area of hypoesthesia after diagnostic sacral lateral branch block. Only 5.4% of patients failed to demonstrate this phenomenon, but all of these patients had a negative block. Hence, the lack of post-injection hypoesthesia in these patients might be attributed to the technical failure of the injection. There is only one other study to our knowledge that investigated the association of a nerve block and RFN with ensuing numbness in the context of assuring the technical adequacy of the procedure [34]. The authors found that 97% of patients undergoing RFN of the third occipital nerve developed hypoesthesia in the suboccipital skin area. Based on this study and previous anatomical dissections reported by Bogduk [35], the current SIS practice guidelines (page 156) recommend checking for post-procedure numbness in the area innervated by third occipital nerve as a means to determine the technical accuracy of this intervention. A lack of numbness indicates technical inadequacy of the procedure [25].

While many factors may influence the success of interventional treatments, one of them - the adequacy and accuracy of the implementation of a procedure by different operators – is almost never discussed because of the lack of instruments available to measure this variable. Based on the previously discussed anatomical studies, SLBs provide skin innervation to the postero-medial buttock through their MCNs subsidiaries; therefore, neurotomy of SLBs theoretically should result in the development of buttock hypoesthesia. However, MCNs may be variable and functionally weak. This means that they do not always become cutaneous or if they do, they are difficult to detect because of the overlapping innervation provided by neighboring nerves. On the other hand, if MCNs are strong and regular, they always have a cutaneous distribution in a sufficiently large area that their function is detectable.

Our study provides compelling data suggesting consistent and clinically detectable buttock skin innervation by MCNs. All the study patients whose sacral lateral branch RFN was successful at 1-month follow-up developed skin hypoesthesia in the area supplied by MCNs. Correspondingly, 77.8% of those who failed neurotomy did not have numbness at that time, strongly suggesting that the technical inadequacy of the procedure could be the reason of the negative outcome in many cases. Two patients were found to have an area of buttock hypoesthesia, even though their procedure was not successful. We hypothesize that in those cases the RFN was performed technically accurately, but the patients' pain did not originate from the SIJC. Even a set of two impeccably performed diagnostic blocks cannot entirely eliminate a placebo response, which could explain such outcomes in these patients. Therefore, the study revealed the strongest association between a lack of post-neurotomy buttock hypoesthesia and failed RFN of SLBs at 1-month follow-up after the intervention. No patients with preserved sensation in the MCNs distribution had a successful RFN and no patients with a successful procedure failed to develop buttock hypoesthesia at that time. At 6 months post-procedure, however, this association disappeared. Two out of five (40.0%) patients who still experienced ≥50% pain relief from the RFN regained full sensation in the previously numb buttock area. Skin re-innervation after nerve injury by expansion of the receptive fields and collateral sprouting of the neighboring undamaged nerves is a well-known phenomenon. This skin re-innervation may occur long before the regeneration of the original damaged nerve [36,37].

Both NRS and ODI scores improved at 1-month follow-up, reaching MCID for the former outcome. More importantly, 57.1% of the patients who underwent RFN of SLBs gained success from this intervention at the primary endpoint of the study. No conclusive data for the secondary outcome measures could be provided for 6-month follow-up because only a few patients were evaluated at that time. Although the success rate of sacral lateral branches RFN in our study is well within the range reported in the literature [[8], [9], [10], [11], [12], [13], [14]], a direct comparison with the other studies would be incorrect because, unlike in the other trials, only those patients with previously failed intraarticular steroid SIJ injections were enrolled in this study. The results of our study buttress the previously reported empirical data [6] indicating that, at least in a proportion of patients, “sacroiliac pain” may arise from structures other than SIJ proper (e.g. posterior ligaments) and is congruent with the studies demonstrating partial SIJ innervation from the ventral rami [38,39].

Our study has certain limitations. We followed the standard neurological sensory examination that does not allow for determination of the magnitude of sensory deficit. Pain sensation was assessed by pinprick rather than by algesimeter, a device occasionally used in special research studies for quantification of sensory deficit(s) [40]. Therefore, we cannot ascertain if all or only some of the SLBs were ablated when an RFN procedure resulted in sensory deficit in the MCNs distribution. Besides, even in case of complete SLB neurotomy, some skin sensation may be preserved because of overlapping receptive fields from neighboring nerves. The related question of whether neurotomy of some but not all SLBs may afford any meaningful pain relief also remains unanswered by our study.

Although only 2 out of 23 (8.9%) patients who underwent RFN were lost to follow-up at the primary end-point of the study, 5 out of 12 whose procedure was successful at 1-month follow-up were not evaluated at 6-month follow-up, primarily due to the COVID-19 pandemic that mostly affected the last stage of the study. Such loss to follow-up lead to our inability to provide meaningful long-term data for the secondary outcome measures. Nevertheless, we believe that validity of the primary outcomes, absence/presence of post-procedural buttock hypoesthesia after diagnostic blocks and at one month after cooled RFN, and the objective of the study were not affected by this attrition.

## 6 Conclusion

The cutaneous subsidiaries of LBSs, middle cluneal nerves, provide regular and clinically detectable innervation to the skin area overlaying postero-medial aspects of the gluteus maximums muscle. Therefore, any technically accurate diagnostic block, irrespective of whether the patients have positive or negative responses, should result in the development of hypoesthesia in the area supplied by the MCNs. Immediately after the completion of the diagnostic procedure, the adequacy of the block should be tested. Absence of hypoesthesia suggests that the block may have been technically inadequate. Numbness in the buttock area innervated by the MCNs may serve as a marker of an adequately performed RFN procedure. If this procedure is unsuccessful in patients who do not develop post-neurotomy numbness in the area supplied by the MCNs, the failure of the intervention may stem from its inaccurate implementation rather than from its inherent ineffectiveness. Instead of being labeled “treatment failures”, such patients might be eligible to have this procedure repeated and, potentially, benefit from it.

## Declaration of competing interest

The authors declare that they have no known competing financial interests or personal relationships that could have appeared to influence the work reported in this paper.
